# Correction: An immunoinformatics and extended molecular dynamics approach for designing a polyvalent vaccine against multiple strains of Human T-lymphotropic virus (HTLV)

**DOI:** 10.1371/journal.pone.0295830

**Published:** 2023-12-08

**Authors:** Abu Tayab Moin, Nurul Amin Rani, Md. Asad Ullah, Rajesh B. Patil, Tanjin Barketullah Robin, Nafisa Nawal, Talha Zubair, Syed Iftakhar Mahamud, Mohammad Najmul Sakib, Nafisa Nawal Islam, Md. Abdul Khaleque, Nurul Absar, Abdullah Mohammad Shohael

The following information is missing from the Acknowledgements section: Our deepest appreciation goes to the International Foundation for Collaborative Research (IFCR), Bangladesh, and the Laboratory of Clinical Genetics, Genomics, and Enzyme Research (LCGGER) at the Department of Genetic Engineering and Biotechnology, University of Chittagong, Chattogram, Bangladesh.

The images for Figs 2 and 3 are incorrectly switched. The image that appears as Fig 2 should be Fig 3, and the image that appears as Fig 3 should be Fig 2. The figure captions appear in the correct order.

**Fig 2:**
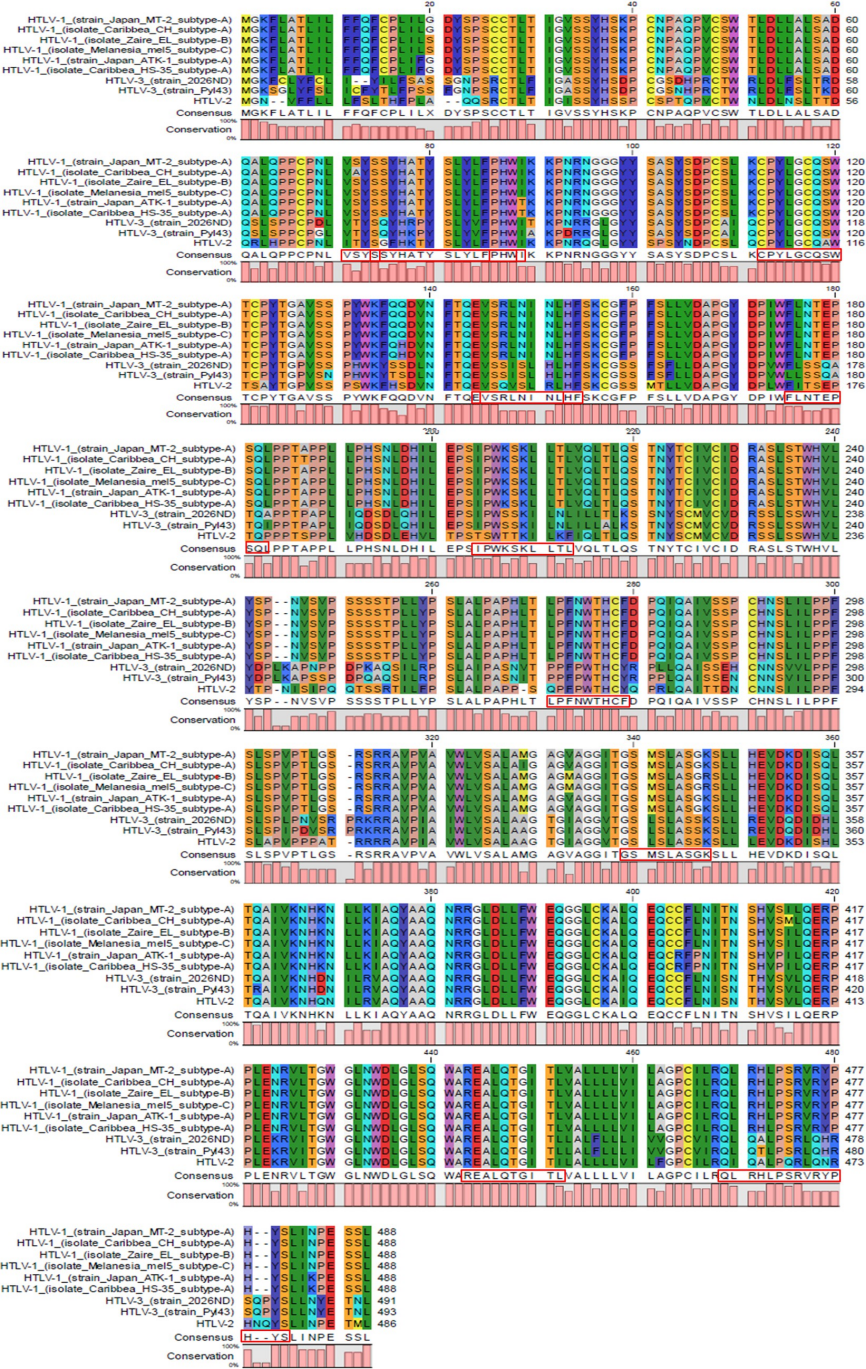
The multiple sequence alignment analysis demonstrating the conservation of the selected epitopes across various strains and isolates of HTLV. The red boxes highlight the presence of these epitopes in multiple HTLV strains, indicating their conserved nature.

**Fig 3:**
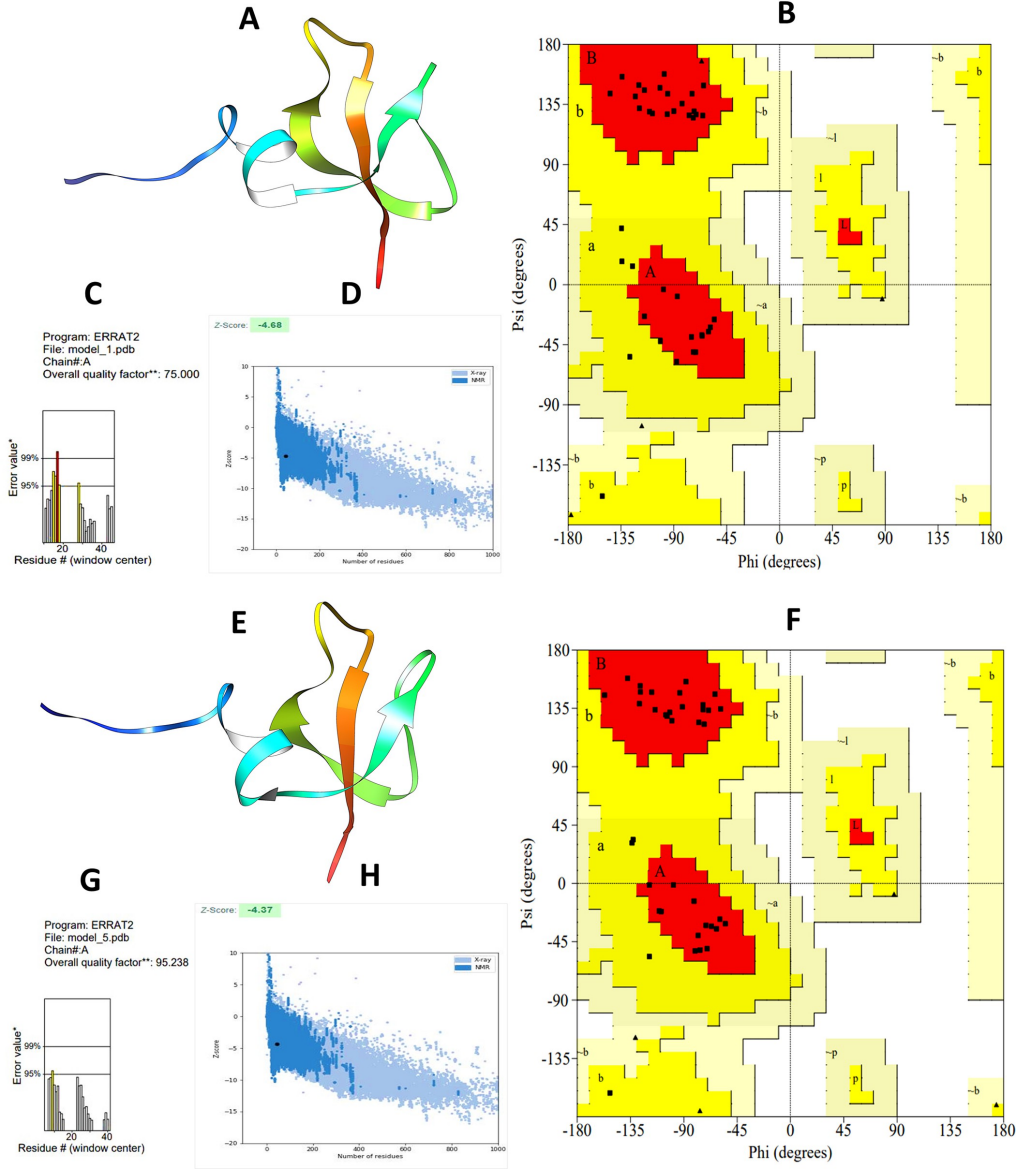
Structure prediction and validation of vaccine construct 1 (A) 3D model (B) Ramachandran layout and (C) The ERRAT quality value (D) Z score graph, and vaccine construct 2 (E) 3D model (F) Ramachandran layout and (G) The ERRAT quality value (H) Z score graph.
